# Ankylosing Spondylitis

**DOI:** 10.18502/jovr.v16i3.9440

**Published:** 2021-07-29

**Authors:** Nazanin Ebrahimiadib, Sahar Berijani, Mohammadreza Ghahari, Fatemeh Golsoorat Pahlaviani

**Affiliations:** ^1^Retina Service, Ocular Immunology and Uveitis Foundation, Farabi Eye Hospital, Tehran University of Medical Sciences, Tehran, Iran; ^2^Retina Service, Farabi Eye Hospital, Tehran University of Medical Sciences, Tehran, Iran

**Keywords:** Ankylosing Spondylitis, Spondyloarthritis, Uveitis

## Abstract

The seronegative spondyloarthropathies are a group of autoimmune inflammatory diseases lacking rheumatoid factor or antinuclear antibody in their serum. They include ankylosing spondylitis (AS), reactive arthritis, psoriatic arthritis, spondylitis associated with Crohn's disease and ulcerative colitis, and undifferentiated spondyloarthropathies. Inflammation mostly affects the axial joints, entheses, and extra-articular structures such as uveal tract, gastrointestinal tract, mucocutaneous tissue, and heart. Uveitis is the most common extra-articular manifestation. Spondyloarthropathies, especially AS, have a strong association with the presence of Human Leukocyte Antigen (HLA)-B27 gene. AS happens earlier in HLA-B27 patients and men are more prone to the disease. Uveitis, typically unilateral non-granulomatous acute anterior uveitis, occurs in up to 50% of the patients with AS. HLA-B27 positivity correlates with more frequent flare-ups. Conjunctivitis and scleritis are rare ocular manifestations of AS. To establish the diagnosis of AS, at least one clinical and one radiologic parameter are required for definitive diagnosis. Magnetic resonance imaging (MRI) or bone scan can help early detection of the axial skeleton inflammation. The course of eye and joint involvement are not correlated. Short-term treatment with topical corticosteroids and cycloplegic agents control the uveitis attack. In resistant cases, local or systemic therapy with corticosteroids are recommended. NSAIDs, disease-modifying anti-rheumatic drugs (DMARDs), methotrexate, azathioprine, anti-IL-17A monoclonal antibodies, and TNF-α antagonists are effective treatments for ocular and systemic manifestations of AS. If not treated adequately, uveitis may become recalcitrant and extend posteriorly. Functional impairment due to joint destruction can also occur as a result of under-treatment.

##  INTRODUCTION

### Origin/History

The seronegative spondyloarthropathies include the following:

• Ankylosing Spondylitis (AS)
• Reactive arthritis (also referred to as Reiter's syndrome)
• Psoriatic arthropathy
• Spondylitis associated with nonspecific inflammatory bowel diseases (IBD) such as Crohn's disease and ulcerative colitis
• Undifferentiated spondyloarthropathies.^[1,2]^

In 1970, physicians described the shared clinical symptoms of the seronegative spondyloarthropathies as a distinct category of diseases distinct from rheumatoid arthritis.^[3]^

When referring to patients with AS (Bechterew disease, Marie-Strumpell disease), they are considered “seronegative” because they typically have a negative rheumatoid factor and antinuclear antibody. Spondyloarthropathy is an umbrella term for a group of rheumatologic diseases that have common clinical features: (1) Inflammation of joints (primarily axial spine and sacroiliac, though peripheral joints may also be affected), (2) Enthesitis, which is defined by inflammation of where tendons, ligaments, and joint capsules are attached to the bone, (3) Extra-articular involvement such as uveitis, gastrointestinal (GI) disease, mucocutaneous lesions and cardiac abnormalities, and (4) the presence of Human Leukocyte Antigen (HLA)-B27 gene.^[2]^

Ocular involvement, especially in the form of anterior uveitis, is the most common extra-articular manifestation of the seronegative spondyloarthropathies.^[4]^ Among the seronegative spondyloarthropathies, AS is known to have the highest association with anterior uveitis.^[1]^ This section will focus on the ocular manifestations of AS.

Methodology: We used PubMed and Google Scholar databases to review the literature. The keywords used were “Spondyloarthritis” AND “Ankylosing Spondylitis” AND “Uveitis”. Out of the 212 results obtained from the search, we selected the articles based on the relevancy to our topic and validity of the studies.

### Epidemiology

AS is the most common form of seronegative spondyloarthropathies with the prevalence of 0.03–1.8%, which varies according to the frequency of HLA-B27 in the population.^[3]^ In the Caucasian population, it ranges between 0.15% and 1.8%. The incidence has been estimated between 0.49 (Japan) and 10 (Norway) per 100,000.^[3]^ Correlation between HLA-B27 and acute anterior uveitis is weakest in African–Americans, intermediate in Asians, and strongest in Whites.^[4]^ Uveitis affects up to 50% of patients with AS while it occurs in approximately 2–5% of patients with inflammatory bowel disease and in about 7% of patients with psoriatic arthritis.^[5]^

Among the seronegative spondyloarthropathies, AS has the strongest association with HLA-B27. Up to 90% of patients with AS have the HLA-B27 haplotype, while this number in the general population is < 10%. Nevertheless, only 1–5% of all HLA-B27-positive individuals will develop the disease, indicating that other genes may play a role in the pathogenesis.^[6,7]^ The association of HLA-B27 and AS disease is, however, less dramatic in non-Caucasians. For example, in a study of a Moroccan population with AS, the HLA-B27 had a frequency of 64%.^[8]^

In terms of clinical presentation, the typical patient with AS is a young man who presents with insidious onset of low back pain and morning stiffness. The disease onset is earlier in HLA-B27 patients. It usually occurs in the second decade of life and rarely occurs after the age of 45. Men are more prone to the disease and they more frequently develop anterior uveitis.^[1]^

### Pathophysiology/Etiology 

The pathogenesis of AS is unknown. However, the trigger of the inflammation may be an immune reaction to an environmental or bacterial antigen in a person with a predisposed genetic background.^[2]^ This may prompt the overexpression of interleukin-12 (IL-12), IL-17, and tumor necrosis factor alpha (TNF-α).^[9,10]^ High levels of TNF-α have been detected in the aqueous and sera of patients with different underlying causes of anterior uveitis including AS.^[2]^

The genetic component, HLA-B27, has been identified as the major predisposing factor for the disease. Like many other HLA class one molecules, it has a high degree of genetic polymorphism. To date, up to 105 subtypes, encoded by 132 alleles, have been identified. The correlation of these subtypes with susceptibility to AS varies. Dominant subtypes that are most commonly associated with the disease are HLA-B*27:05 (Caucasians), HLA-B*27:02 (Mediterranean populations), and HLA-B*27:04 (Chinese).^[11]^ HLA-DRB1 alleles have been also identified as another genetic risk factor for AS. HLA-DRB1*08 positivity is associated with higher levels of TNF-α in the aqueous humor of patients with active uveitis.^[11]^

### Definition and Criteria for Diagnosis

The modified New York Criteria described in 1984 outlines the diagnostic criteria for AS. It is based on clinical manifestations and evidence of sacroiliitis on X-ray. At least one clinical and one radiologic parameter are required for definitive diagnosis of AS.^[1]^ The clinical manifestations/parameters include: (1) history of inflammatory pain and morning stiffness in the lumbar spine for at least three months that improves with exercise and is not relieved by rest, (2) limitation of lumbar spine motion in both frontal and sagittal planes, and (3) limitation of chest expansion compared to normal values for age and gender. The radiographic parameters include X-ray evidence of grade two to four sacroiliitis bilaterally or grade three or four sacroiliitis unilaterally.^[12]^ Sclerosis and obliteration of the joint space, ligamentous calcification, squaring of the vertebrae, and in end stage disease, the characteristic ankylosed “bamboo” spine can be seen on plain radiography. It is noteworthy that seropositivity is neither necessary nor sufficient for establishing the diagnosis of AS.^[13]^

Establishing the diagnosis of AS relies on a thorough history and physical examination besides radiologic confirmation. The disease should not be ruled out if the aforementioned criteria are not met.^[2]^ Radiologic evidence of lumbar spine involvement may not appear in the first year of disease onset, so probable diagnosis of AS in the presence of typical clinical manifestation must continue to be a consideration. Other risk factors that may help to establish an earlier diagnosis are: absence of rheumatoid factor, HLA-B27 seropositivity, family history, male gender, disease onset prior to the age of 40 years, and frequent gastroenteritis.^[2,14]^

During the Annual Scientific Meeting of the American College of Rheumatology in 2009, new criteria for the diagnosis of AS were discussed and magnetic resonance imaging (MRI) was included to aid with the diagnosis of axial skeleton inflammation.^[2]^ This imaging modality has been proven to be more sensitive in detecting joint inflammation many years earlier than conventional radiography. With earlier diagnosis and treatment, permanent cartilage damage and bony erosions causing spinal deformity can be prevented.^[15,16]^ Therefore, it is of utmost importance to suspect AS in patients with a history of inflammatory spinal pain or recurrent attacks of anterior uveitis. If plain radiography is negative in situations of high clinical suspicion, MRI and HLA-B27 testing should be requested. If MRI is contraindicated, bone scan can also be helpful in diagnosis of AS if plain films are normal.^[15,16]^

Laboratory findings in patients with AS may serve as markers of chronic disease but are not specific or diagnostic. These include normochromic and normocytic anemia, mild leukocytosis, increased erythrocyte sedimentation rate (ESR), increased C-reactive protein (CRP), and elevations in alkaline phosphatase and IgA.^[2]^

### Symptoms and Signs

#### Systemic Inflammation

Systemic inflammation primarily affects the sacroiliac joint, the spine, and the entheses. The patient may feel a unilateral or intermittent pain in the gluteal region or the lumbosacral area that within a few months becomes persistent and bilateral. In advanced disease, chronic and progressive inflammation leads to fusion of the sacroiliac joint and spine with progressive loss of spinal movement, loss of lumbar lordosis and kyphosis, and restricted respiratory excursion. “Bamboo spine” is a radiologic sign for an advanced disease. In addition, enthesopathy, such as Achilles tendonitis, plantar fasciitis, intercostal muscle tendonitis, and dactylitis can occur early in the disease and are painful and recurrent, resulting in structural damage. Other peripheral joints such as knee, hip, and shoulder can also be involved, typically as an asymmetric oligoarthritis that predominantly affects the lower limbs.^[2]^

Rare extra-articular manifestations of the disease include upper lobe pulmonary fibrosis, cardiac involvements, aortic regurgitation, chronic prostatitis, cauda equina syndrome, occult bowel lesions, and amyloid deposition. These manifestations may present years after active disease.^[2]^ Patients may also present with constitutional symptoms including low grade fever, anorexia, fatigue, and weight loss.^[2]^

#### Ocular Manifestations 

Acute anterior uveitis is the most common non-articular manifestation of AS and presents in up to 30% of patients during the course of the disease and may increase to 50% with longer follow-up.^[4]^ Uveitis is most often characterized by recurrent, asymmetric and bilateral iridocyclitis, involving only one eye at a time and is not related to the severity and course of joint involvement.^[17]^ It may be the first manifestation of AS, preceding the other articular symptoms. Underlying AS is diagnosed in about 24.3% of patients presenting with idiopathic acute anterior uveitis.^[18]^ As a result, ophthalmologists play an important role in early diagnosis and treatment of the disease, which can lead to a more favorable prognosis.^[19]^ HLA-B27 positivity correlates with more frequent relapses and worse prognosis of uveitis. The interval between attacks is highly variable and can range from one month to 35 years, although most commonly they occur between 14 and 25 months.^[4]^

Common forms of anterior uveitis in AS are iritis (with inflammatory cells in the anterior chamber and no involvement of the anterior vitreous), iridocyclitis (primary inflammation of the iris and secondary inflammation of the ciliary body; inflammatory cells present in both the anterior chamber and anterior vitreous), and cyclitis (inflammation of mainly the ciliary body).^[2]^ Patients typically present with sudden ocular pain, redness, photophobia, and decreased vision.^[20]^ The main findings on examination are limbal injection, fine whitish–gray keratic precipitates, moderate to severe amounts of cells which may sometimes cause hypopyon and fibrinous exudate in the anterior chamber.^[5]^ Inflammatory cells in the anterior chamber of patients with AS are more static in nature. Thus, hypopyon seen in patients with AS can be distinguished from hypopyon that shifts easily in patients with Bechet's disease.^[21]^ Although, non-granulomatous anterior uveitis is the typical manifestation, patients may present with posterior uveitis (choroiditis or retinochoroiditis), intermediate uveitis (vitritis, peripheral retinitis, and pars planitis), or panuveitis.^[1,4]^

Initial therapies include topical corticosteroids and mydriatic drugs to avoid posterior synechia.^[2]^ If not treated properly, inflammation can extend to the posterior segment of the eye and vitritis, papillitis, retinal vasculitis, cystoid macular edema, epiretinal membrane, and pars plana exudate can occur.^[22]^

Conjunctivitis and scleritis are other less common ocular manifestations of AS. Conjunctivitis is often bilateral, non-purulent, and self-limited. Scleritis may occur early or years after the disease activation.^[2]^

### Disease Monitoring 

There is no individual blood marker to measure disease activity. Monitoring of the disease is subjective and is based on the clinical assessment. Functional deterioration can help monitor disease activity.^[23]^ Ophthalmologists can schedule the frequency of follow-up visits based on the number and severity of uveitis episodes per year.

Radiographic imaging such as contrast enhanced MRI of the sacroiliac joint helps in evaluating joint damage and gives further information about ongoing inflammation. This information in addition to measuring serologic acute phase reactants, such as CRP and ESR, may be useful in monitoring disease activity and the potential for further damage to the sacroiliac joint.^[23]^

### Risk Factors

#### Acquired

Acquired risk factors that have been proposed include previous urogenital or GI infections. Previously implicated bacteria include Chlamydia trachomatis, gram-negative enterobacteria including Klebsiella, Salmonella, Yersinia, Shigella, and Campylobacter jejuni.^[2]^ Patients with spondyloarthropathy, especially male gender, have increased colonization of these bacteria and subsequently higher titers of related antibodies. Colonization of Klebsiella in the bowel has been shown to be associated with higher number of anterior uveitis episodes in these patients.^[14]^

#### Genetic

The most important factor is the presence of HLA-B27 on the short arm of chromosome six. However, other genes that are believed to have a role in this disease are HLA-B60, HLA-B61, HLA-DR8, HLA-DRB1, and MICA (MHC class I chain-related gene A).^[2][6,7][9][11]^

### Treatment

Acute episodes of anterior uveitis associated with AS respond well to frequent topical corticosteroids and cycloplegic agents for a short period of time. Most patients recover full vision within two months of resolution of a flare.^[4]^ Delayed or insufficient treatment makes uveitis more recalcitrant to therapy. Approximately 13–19% of patients are resistant to topical therapies, and often the disease in these patients becomes chronic.^[2]^ If topical therapies alone are not effective, periocular injection of triamcinolone (40 mg/ml), intraocular injection of corticosteroids, or a short course of systemic corticosteroid therapy may be necessary, particularly in cases complicated by cystoid macular edema.^[1]^

Non-steroidal anti-inflammatory drugs (NSAIDs) or coxibs alleviate symptoms of inflammatory back pain and may have positive effects on uveitis attacks and provide the opportunity for steroid-free remission.^[24]^ Coxibs carry less risk of GI side-effects than NSAIDs and can be used in patients who are intolerant to NSAID therapy.^[24]^

Disease-modifying Anti-Rheumatic Drugs (DMARDs) are a group of medications that are effective in inducing remission in AS. Sulfasalazine works by inhibiting the synthesis of prostaglandins. It has been used in recurrent acute anterior uveitis in AS, especially in patients with peripheral arthritis.^[2]^ It has been shown to reduce the number and severity of uveitis relapses.^[20]^ Uveitis that is refractory to sulfasalazine may benefit from steroid-sparing immunosuppression which can decrease the number of flares. Methotrexate (7.5–25 mg), dosed weekly, and azathioprine (1–2 mg/kg), dosed daily, are two examples of immunosuppressive therapy that may be used for the uveitis associated with AS.^[25]^

Methotrexate is considered an anti-metabolite and works by competitively inhibiting dihydrofolate reductase (DHFR), ultimately interfering with DNA synthesis. The role of methotrexate on the natural course of uveitis is conflicting in the literature. Some studies report that it can reduce the number of uveitis relapses, while others refute its ability to modify the disease or reduce the uveitis crisis.^[2,25,26]^ Patients taking methotrexate or azathioprine must be monitored with questions about side effects and frequent blood tests.^[25]^

In cases refractory or intolerable to NSAIDs, sulfasalazine and immunosuppressant drugs or in severe disease with extension of inflammation into the posterior segment, anti-TNF-α therapy is indicated.^[27]^ Some authors even suggest that after failure of two trials of NSAIDs for more than three months or intolerable side-effects, therapy with TNF-α inhibitors should be initiated.^[27]^

Biologic agents, including TNF-α inhibitors, have revolutionized the treatment of spondyloarthropathies as they have been shown to modify the course of the disease and significantly reduce the rate of uveitis recurrences.^[4]^ TNF-α inhibitors consist of four monoclonal antibodies (adalimumab, certolizumab, golimumab, and infliximab) and one TNF-receptor fusion protein (etanercept). By binding to TNF-α, they prevent it from binding to lymphocyte Fc receptors. Therefore, cellular immunity is suppressed. In addition to their rapid onset of action and promising results in modifying the inflammatory course of the disease, this class of medications has substantially contributed to improved joint mobility and visual recovery,^[28,29]^ and can be used as monotherapy.^[2]^

All four TNF-α antagonists have been proven to be equally effective in controlling the spinal manifestations of AS and uveitis; however, infliximab and adalimumab have been shown to be slightly more effective for the treatment of extra-spinal features of the disease including acute uveitis. These two agents, especially infliximab, significantly reduce the number of uveitis flare-ups.^[30]^ However, their high cost and potential complications prevent them from being used as the first-line therapy in uveitis. Etanercept, although in the same overall class of medications, is not efficacious in the treatment of ocular inflammation and has been reported to paradoxically cause uveitis.^[10]^

Some potential side-effects of TNF-α antagonists include infection (particularly tuberculosis and histoplasmosis), exacerbation of demyelinating diseases, bilateral anterior optic neuropathy, and sudden death in patients with congestive heart failure.^[2]^

Anti-IL-17 monoclonal antibodies have been approved for the treatment of AS; however, they are mostly effective for the joint manifestations rather than ocular inflammation.^[32]^

Functional impairment and pain are the two major areas that should also be addressed in the treatment of arthritis in AS. Depending on the joint involved, patients with AS need to undergo physical therapy to improve function and mobility. Patient education is critical. Posturing exercises, local heat, and job modification can help patients to maintain muscle strength and flexibility even with progression of ossification and ankyloses.^[33]^

Similar to other types of uveitis, patients may develop complications such as glaucoma and cataract which require surgical management.^[34]^ Trabeculectomy and Ahmed valve procedure are the most common surgeries performed for glaucoma, although valve surgery is usually preferred due to the propensity of these patients to scar.^[35]^ Cataract extraction is often complex. Patients often have small pupils secondary to posterior synechia requiring lysis and pupil-dilating devices at the time of surgery. Before proceeding with cataract surgery, the patient must be free of inflammation for at least three months and the surgeon should counsel the patient about the need for frequent corticosteroid drops and/or oral corticosteroids as well as topical NSAIDs before and after surgery.^[36]^ In rare cases of extension of inflammation to the vitreous cavity or vitreomacular traction, vitrectomy may be required.^[37]^

### Prognosis

Long-term treatment with anti-inflammatory drugs is usually required in patients with AS. The goal of treatment is preserving the high quality of life with maximum function. Some risk factors for poor prognosis are as follows: involvement of peripheral joints, disease onset during youth, endless steroid therapy, and poor response to NSAIDs.^[2]^

The prognosis of uveitis in AS is usually good except for refractory cases with involvement of the posterior segment. Most patients regain full vision in two months.^[4]^ Unfortunately, delayed and ineffective therapy of patients is very common. HLA-B27 positivity is associated with higher frequency of uveitis recurrence and therefore with worse visual prognosis.^[4]^ Early stages of sacroiliitis can be detected in about 63% of patients with acute anterior uveitis using sophisticated techniques of imaging such as bone scan.^[30]^ This may allow a prompt diagnosis and earlier effective care.

The prognosis of joint involvement is also good; however, intermittent flare-ups between bouts of clinical remission can cause complete spinal ankylosis. Vertebral fusion, which includes the cervical region, results in severe kyphosis and limited mobility. This makes patients vulnerable to fracture even with trivial trauma. Hip and shoulder involvement require total joint replacement.^[2]^ These are important signs that the ophthalmologist should pay attention to in the evaluation of their patients.

Some patients remain in the “non-radiographic” phase for years. The major predictor of progression of disease from non-radiographic to radiographic stage is the amount of inflammation. Therefore, in patients with an elevated CRP and evidence of active inflammation in the sacroiliac joint detected by MRI, structural damage of the sacroiliac joint is more likely to occur.^[19,31]^

### Complications 

Complications can occur secondary to chronic and recurrent episodes of uveitis or delayed and insufficient treatment. Posterior synechia is the most common anterior segment complication (13–91%) followed by cataract (7–28%). This complication can make cataract surgery more complicated. Glaucoma, diffuse vitritis (the most common form of posterior segment involvement), as well as cystoid macular edema (the most common cause of visual impairment) are often observed.^[18]^

Cervical spinal subluxation, aortic regurgitation, respiratory failure, and amyloidosis are rare potentially fatal systemic complications of the disease.^[2]^

### Future Directions 

NSAIDs and TNF-α antagonists are two mainstays of therapy in AS. TNF-α antagonists are the most effective agent in controlling inflammation, however, about 20–30% of patients are unresponsive to this class of drugs. Other new biologic response modifiers that target IL-23 or IL-17, especially IL-17, are being considered as an alternative to anti-TNF-α antagonists in treatment of seronegative spondyloarthropathies. Secukinumab is an example of anti-IL-17A monoclonal antibody that has shown promising results in these patients.^[32]^ New therapies that may provide functional relief to patients are on the horizon. Further studies are expected to include ocular manifestations of the disease, as these can cause significant morbidity in patients with ankylosing spondyloarthropathies.

##  Financial Support and Sponsorship

Nil.

##  Conflicts of Interest

There are no conflicts of interest.
